# Improvement in the transport critical current density and microstructure of isotopic Mg^11^B_2_ monofilament wires by optimizing the sintering temperature

**DOI:** 10.1038/srep36660

**Published:** 2016-11-08

**Authors:** Wenbin Qiu, Hyunseock Jie, Dipak Patel, Yao Lu, Vladimir Luzin, Arnaud Devred, Mehmet Somer, Mohammed Shahabuddin, Jung Ho Kim, Zongqing Ma, Shi Xue Dou, Md. Shahriar Al Hossain

**Affiliations:** 1 Institute for Superconducting and Electronic Materials, Australian Institute for Innovative Materials (AIIM), University of Wollongong, Squires Way, North Wollongong, NSW 2500, Australia; 2 Australian Nuclear Science Technology Organisation (ANSTO), Lucas Heights, NSW 2232, Australia; 3 ITER Organization, 13115 Saint Paul Lez Durance, France; 4 Koc University, Chemistry Department, Rumelifeneri Yolu, TR-34450 Sariyer-Istanbul, Turkey; 5 Department of Physics and Astronomy, College of Science, King Saud University, Riyadh 11451, Saudi Arabia

## Abstract

Superconducting wires are widely used in fabricating magnetic coils in fusion reactors. In consideration of the stability of ^11^B against neutron irradiation and lower induced radio-activation properties, MgB_2_ superconductor with ^11^B serving as boron source is an alternative candidate to be used in fusion reactor with severe irradiation environment. In present work, a batch of monofilament isotopic Mg^11^B_2_ wires with amorphous ^11^B powder as precursor were fabricated using powder-in-tube (PIT) process at different sintering temperature, and the evolution of their microstructure and corresponding superconducting properties was systemically investigated. Accordingly, the best transport critical current density (*J*_*c*_) = 2 × 10^4^ A/cm^2^ was obtained at 4.2 K and 5 T, which is even comparable to multi-filament Mg^11^B_2_ isotope wires reported in other work. Surprisingly, transport *J*_*c*_ vanished in our wire which was heat-treated at excessively high temperature (800 °C). Combined with microstructure observation, it was found that lots of big interconnected microcracks and voids that can isolate the MgB_2_ grains formed in this whole sample, resulting in significant deterioration in inter-grain connectivity. The results can be a constructive guide in fabricating Mg^11^B_2_ wires to be used as magnet coils in fusion reactor systems such as ITER-type tokamak magnet.

Fusion power is one of the most promising candidate energy sources that may solve global energy problems, considering its safer and greener merits compared with the conventional mineral energy sources. In the world-class International Thermonuclear Experimental Reactor (ITER) fusion energy project, the superconducting magnet system serves as a key determinant. A high and steady magnetic field needs to be produced to confine the deuterium (D)–tritium (T) burning plasma inside the ITER tokamak nuclear fusion reactor. According to the previous ITER plan, hundreds of tons of superconducting magnets made from NbTi and Nb_3_Sn will be fabricated to assemble 18 Nb_3_Sn toroidal field (TF) coils, a 6-module Nb_3_Sn central solenoid (CS) coil, 6 Nb-Ti poloidal field (PF) coils, and 9 pairs of Nb-Ti correction coils (CC)^1^,^2^. There is one major drawback, however, for the application of Nb-based superconductors in this project. After irradiation, ^93^Nb will be transformed into the long-lived nuclide ^94^Nb with a half-life of about 20,000 years^3^,^4^. Hence, before irradiated Nb-based alloys are safe to be recycled, tens of thousands of years are required for them to “cool down”, and meanwhile, thicker shielding is necessary for long-term operation. For the convenience of radioactive waste treatment and environmental protection, the radioactivation properties of superconducting components within the fusion reactor should be taken into account. Compared with conventional Nb-based superconductors, MgB_2_ features “low activation” and a much shorter decay time. Within 1 year, the dose rate of MgB_2_ materials will be reduced to the hands-on maintenance level, which is considered as desirable for a fusion reactor magnet system^3^. Additionally, because of the reaction ^10^B + n → 7Li + He (gas) under the heavy irradiation condition, ^10^B can no longer guarantee the stability of the MgB_2_ superconducting magnet. By replacing ^10^B with the isotope ^11^B, Mg^11^B_2_ superconducting wires will be much more stable in a neutron irradiation environment due to the smaller neutron capture cross-section of ^11^B^5^. Considering the abundant reserves of ^11^B on Earth (20 wt% for ^10^B, 80 wt% for ^11^B), the anticipated cost for extracting the isotope from natural boron is expected to be decreased during the chemical synthesis.

The superconductivity of MgB_2_ was discovered in 2001^6^. It is well-known for its simple binary chemical composition and much higher critical transition temperature (*T*_*c*_) of 39 K than that of NbTi at 9.3 K. In order to operate Nb-based low-temperature superconductors, the core of the magnet needs to be cooled down to 4 K. The only eligible cryogen is liquid helium, which is extremely expensive, not always available on hand, and very difficult to handle. In the case of MgB_2_, a working temperature as high as 20 K is low enough to achieve acceptable performance. Remarkably, the operating cost is expected to be cut by over 50% by substituting cryocooler-cooled MgB_2_ materials for liquid-helium-cooled Nb-based superconductors. Furthermore, the fabrication cost of MgB_2_ superconducting wire itself ($2.64/kA∙m) is less than 1/3 of that of Nb_3_Sn wire ($9/kA∙m). Therefore, due to the advantages of cost-effectiveness, lower radioactivation, and the shorter decay time of isotopic Mg^11^B_2_, fundamental research on Mg^11^B_2_ superconducting wires will be valuable for improving the efficiency of practical application in high-irradiation environments such as fusion reactors.

Mg^11^B_2_ wires using isotopically pure ^11^B powder always show lower critical current density (*J*_*c*_) values, however, than the wires fabricated with natural boron powder. According to previous work^7^,^8^, this lower *J*_*c*_ is a result of the increased amount of non-reactive precursor, which decreases the superconducting fraction. On the other hand, inter-grain connectivity is considered another crucial factor in the current-carrying capability of Mg^11^B_2_ superconducting wires^9^,^10^,^11^. In this work, with the aim of further improving *J*_*c*_ in Mg^11^B_2_ wires, the evolution of the microstructure and superconducting performance in Mg^11^B_2_ wires sintered at different temperatures was investigated in detail. The influence of both the superconducting fraction and the inter-grain connectivity on the *J*_*c*_ performance is discussed. We optimized the temperature of the heat-treatment at which the best transport performance can be obtained. Surprisingly, in the case of Mg^11^B_2_ wire sintered at high temperature, the transport *J*_*c*_ vanished, although magnetic *J*_*c*_ was still detected. According to detailed microstructure observations, this could be ascribed to the formation of a unique microstructure that was only obtained in the sample sintered at excessively high temperature. This kind of microstructure leads to significant deterioration in inter-grain connectivity and ultimately, poor transport current performance.

## Experimental Details

The standard *in-situ* powder-in-tube (PIT) procedure was applied to all the samples. The starting materials for the Mg^11^B_2_ wire consisted of ^11^B amorphous powder (from Pavezyum Kimya, Turkey, Moissan method^12^, 95.5%) and magnesium powder (100–200 mesh, 99%). The isotopic purity and particle size with respect to the ^11^B enriched boron powder was >99.5% and 840 nm, respectively. After mixing the precursor powders, the mixture was tightly packed into Nb/Monel tubes with 10 mm outer diameter and 6 mm inner diameter. The composite wire was swaged and drawn to a final outer diameter of 1.08 mm. Then, the fabricated Mg^11^B_2_ wires were sintered at different temperatures ranging from 700 °C, 750 °C, 770 °C, and 800 °C for 60 min (ramp rate: 5 °C/min) under high purity flowing argon gas. Finally, the samples were furnace-cooled to room temperature.

The transport critical current (*I*_*c*_) measurements were carried out by using an American Magnetics superconducting magnet with DC current (with the upper limit of the current source 200 A) under possible magnetic field up to 15 T, with the standard four-probe method and the criterion of 1 μV/cm. The critical current density *J*_*c*_ was calculated by dividing *I*_*c*_ by the cross-section of the Mg^11^B_2_ core, which was examined with an optical microscope (Leica M205A). Scanning electron microscopy (SEM, JEOL JSM-6490LV & JEOL JSM-7500) was employed to observe the microstructure under different magnifications. X-ray diffraction (XRD) *θ–2θ* scans (GBC-MMA) were used to identify the phase composition. Measurements of electrical resistivity and magnetic moment were conducted in a 9 T Physical Properties Measurement System (PPMS, Quantum Design). In case of XRD, SEM, and PPMS measurements, the outer sheaths of the Mg^11^B_2_/Nb/Monel wires were removed for better data accuracy.

## Results and Discussion

**Figure 1:**
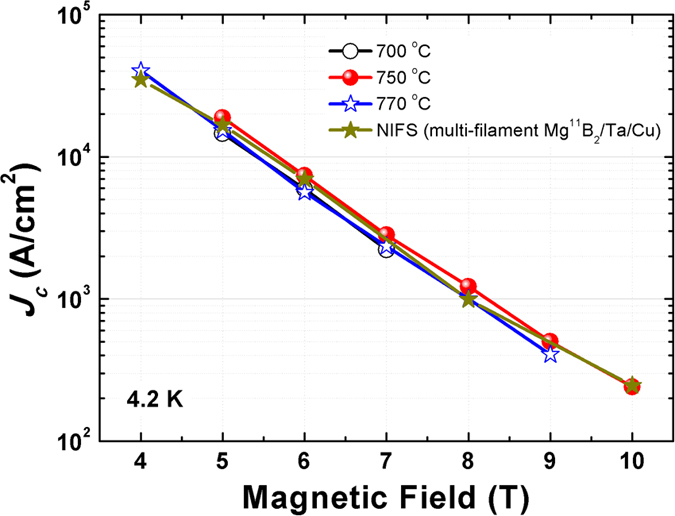
Transport *J*_*c*_ - *B* performance at 4.2 K of Mg^11^B_2_ wires using amorphous ^11^B isotope as the boron source. Results from NIFS are also plotted for reference. No transport *I*_*c*_ was detected in the wire treated at 800 °C.

Typical transport *J*_*c*_ - *B* performances of all four wires sintered at different temperatures are shown in Fig. 1. For reference purposes, transport *J*_*c*_ data of for the multi-filament Mg^11^B_2_/Ta/Cu wire reported by Hishinuma^7^ is also plotted in the figure. It should be noted that our best monofilament Mg^11^B_2_ wire shows comparable transport *J*_*c*_ performance to the multifilament wire fabricated by the National Institute for Fusion Science (NIFS)^7^. This result is considered as a big breakthrough, and it strongly supports the feasibility of replacing commercial NbTi by high-performance Mg^11^B_2_ wires in highly radioactive fusion reactors. In our Mg^11^B_2_ wires, 750 °C is the optimized temperature for heat treatment. The corresponding wire possesses a *J*_*c*_ value near 2 × 10^4^ A/cm^2^ at 4.2 K and 5 T. Slight *J*_*c*_ degradation is observed in the wire treated at temperatures deviating from 750 °C. Surprisingly, no transport current was detected in the wire treated at 800 °C. For verification, five attempts at measurement were carried out on three batches of wires produced under the same sintering conditions. Ultimately, none of them gave detectable transport current data. It is speculated that some unexpected qualitative change inside the wire might occur once the heating temperature reaches a certain level. This should probably be attributed to a unique property of the ^11^B starting powder. It is believed that investigations of the phase composition, microstructure, and inter-grain connectivity will give an explanation for this abnormal phenomenon.

**Figure 2:**
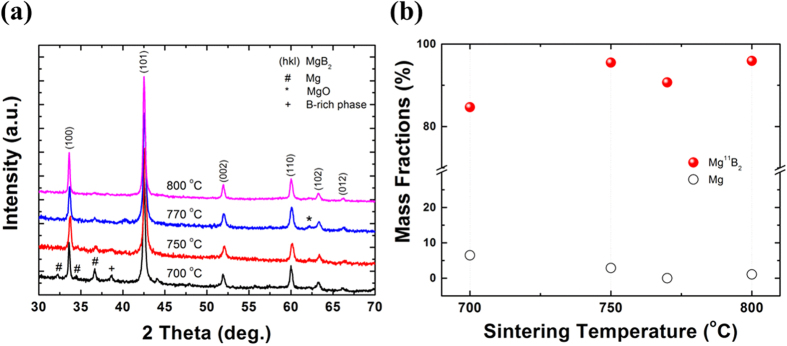
(**a**) XRD *θ–2θ* patterns of all Mg^11^B_2_ wires sintered at different temperatures. The numbered labels (hkl) represent Mg^11^B_2_ reflections. The pound sign (hashtag) stands for unreacted Mg. A small amount of B-rich phase (with its peak marked by the plus sign) is detected only in samples sintered at 700 °C. (**b**) Mass fractions, obtained from Rietveld refinement, of Mg^11^B_2_ and Mg as functions of the different sintering temperatures.

To confirm the phase composition, Mg^11^B_2_ cores were removed from their outer sheaths and finely ground as XRD specimens. In Fig. 2(a), the main peaks indexed as Mg^11^B_2_ can be observed in all spectra, indicating that the temperature is high enough to permit the formation of Mg^11^B_2_ phase. Very little oxidation was detected, according to the negligible MgO peak. Un-reacted Mg and ^11^B-rich phase are found in the wire sintered at relatively low temperature. Apparently, it is very hard for Mg to completely diffuse into boron particles, if the sintering temperature is not high enough. A diminishing gradient of Mg concentration exists along the radial direction of the boron particle. As a result, Mg^11^B_2_ phase can only be formed on the outer layers of boron particles. The rest of the Mg will either stay in the elemental state (un-reacted Mg) or participate in other secondary reactions. Hence, ^11^B-rich phase is prone to form in this case, which can be deduced from the Mg-B phase diagram^13^. The presence of those impurities (un-reacted Mg and ^11^B-rich phase) will reduce the fraction of superconducting phase, which is crucial for the final performance of superconductors. It has to be pointed out that the chemical activity of ^11^B is lower in comparison with natural B due to the isotope kinetic effect^14^. This might explain why 700 °C is not high enough for the complete reaction in this work. Figure 2(b) shows the mass fractions of Mg^11^B_2_ phase in the wires as a function of sintering temperature. The mass fractions were calculated by using Rietveld refinement. The smallest Mg^11^B_2_ fraction, as low as 84.7%, is found in the wire treated at 700 °C. This is mainly due to the presence of impurities, as reflected by the XRD results. Furthermore, the degradation in transport *J*_*c*_ performance also confirms its relatively poor superconductivity (see Fig. 1). With increasing sintering temperature, un-reacted Mg peaks become smaller and almost disappear. Correspondingly, the mass fractions of Mg^11^B_2_ phase in the rest of the wires all remain at a high level (>90%). Since the crystallization of Mg^11^B_2_ phase is confirmed to be good in the Mg^11^B_2_ wire sintered at 800 °C, while its mass fraction of superconducting phase is also satisfactory, the observed abrupt disappearance of transport current in the wire sintered at 800 °C is related to neither the phase composition nor a low superconducting fraction.

**Figure 3:**
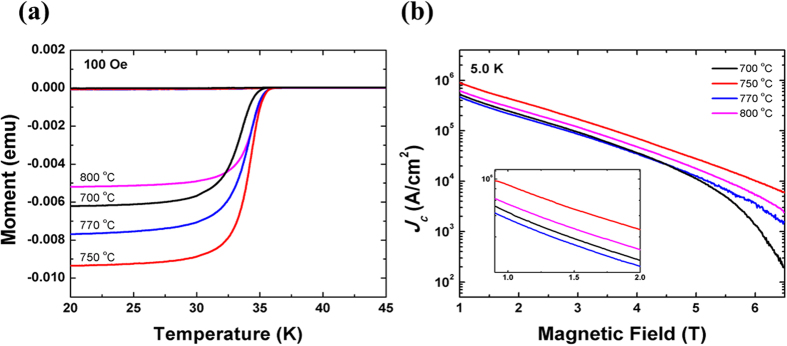
(**a**) Temperature dependence of the ZFC and FC dc magnetization measured in a field of 100 Oe. (**b**) Field dependence of the magnetic *J*_*c*_ at 5.0 K on the logarithmic scale (with the inset showing an enlargement of the *J*_*c*_ (*H*) at low fields) for all samples.

Figure 3(a) shows the zero-field-cooled (ZFC) and field-cooled (FC) demagnetization results as functions of the sintering temperature for all four samples. *H* = 100 Oe was applied in this measurement. A clear normal-superconducting transition was observed in all samples, including the wire sintered at 800 °C, which did not show any current value in the transport measurements. The magnetic *J*_*c*_ (*H*) of the samples was estimated at 5 K based on the magnetization hysteresis loops and the Bean critical state model. Generally, the formula for a rectangular shaped sample is: *J*_*c*_ = 20(Δ*M*/*V*)/[*a*(1−*a*/3*b*)], where Δ*M* = [*M*(+) − *M*(−)] is the difference between the upper and lower branches of the *M*(*H*) loop, *V* is the volume, and *a* and *b (a* < *b*) are the length and width of the cross-section which is perpendicular to the direction of the applied magnetic field^15^. In our case, the Mg^11^B_2_ cores are cylindrical in shape. So, the formula can be simplified to *J*_*c*_ = 30(Δ*M*/*V*)/*d*, where *d* is the diameter of the circular cross-sectional area^16^,^17^. According to the calculations, the magnetic *J*_*c*_ (*H*) results at 5.0 K are shown in Fig. 3(b). The wire sintered at 750 °C shows the best magnetic *J*_*c*_ (*H*) performance throughout the entire range of fields, which is consistent with the transport *J*_*c*_ results shown in Fig. 1. Some differences can be found between the values of magnetic *J*_*c*_ and transport *J*_*c*_. Other than measurement deviation, the intrinsic distinction between the magnetic *J*_*c*_ signal and the transport *J*_*c*_ signal also needs to be taken into consideration. Generally, due to the existence of negative structures such as porosity and cracks, not all the MgB_2_ in a sample is capable of passing transport current. Inter- or intra-grain connectivity should always be considered when dealing with transport performance. On the contrary, as long as they possess superconductivity, all the MgB_2_ fragments will contribute to the magnetic *J*_*c*_. It should be noted that the magnetic *J*_*c*_ was detected and showed good performance in the wire sintered at 800^ ^°C. This means that the Mg^11^B_2_ superconducting phase in the wire was not badly damaged by the high sintering temperature. Hence, after ruling out the effects of inferior superconducting phase, it can be speculated that the transport current in the wire sintered at 800 °C disappeared as a result of a problem with inter-grain connectivity. A high sintering temperature might introduce some defects and significantly destroy the connection between Mg^11^B_2_ superconducting grains.

**Figure 4:**
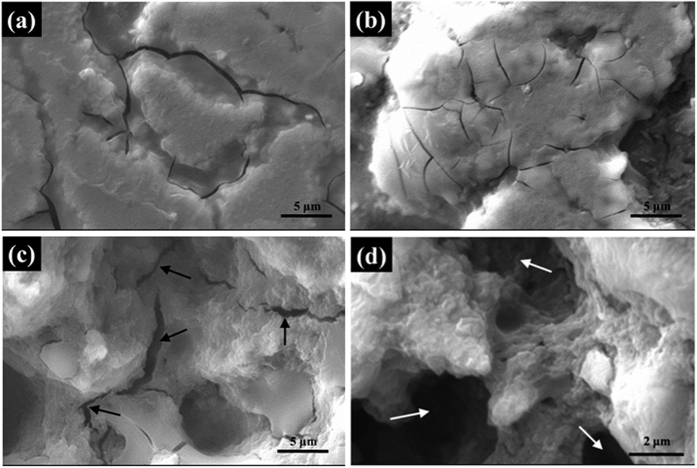
SEM micrographs of cross-sections of Mg^11^B_2_ wires sintered at (**a**) 700 °C, (**b**) 750 °C, and (**c**) 800 °C. Evolution of the surface morphology is clearly shown. Black arrows indicate big cracks. (**d**) SEM image of the wire sintered at 800 °C under higher magnification. White arrows indicate porous structure in the sample sintered at 800 °C.

It is estimated that the vanishing of transport current in the Mg^11^B_2_ wire is caused by the severe deterioration of inter-grain connectivity, which can be visually confirmed by SEM micrographs. The low-magnification SEM images of the cross-sections of Mg^11^B_2_ wires sintered at 700 °C, 750 °C and 800 °C are presented in Fig. 4(a–c). Obvious evolution of the surface morphology is exhibited with increasing temperature. In the wire treated at 700 °C, it was already proved by the XRD results that the Mg had partially reacted with the boron. As the particle size of the Mg powder is much bigger than for the boron powder, un-reacted Mg melted and smoothly covered the Mg^11^B_2_ grains. Therefore, the morphology of this sample was fairly plain and incompact. A dense surface is observed in Fig. 4(b) on the optimal sample sintered at 750 °C, indicating complete reaction and good inter-grain connectivity. This is consistent with the *J*_*c*_ - *B* and XRD results discussed above. Once the sintering temperature reached 800 °C, big cracks (marked by black arrows) were observed, as shown in Fig. 4(c). They are much bigger than the normal microcracks in other samples. Note that most of the big cracks are connected with each other. This feature is considered to be highly detrimental to the inter-grain connectivity. The resultant superconducting fragments are isolated from each other, and eventually, very little current can pass through the entire wire, which will significantly reduce the transport performance. On further increasing the magnification, porous structure is found in the same sample (marked by white arrows in Fig. 4(d)). When the wire was heat-treated at 800 °C, both the grain size and the mobility of the Mg^11^B_2_ grains were increased. The separate grains are prone to aggregate with each other, leaving plenty of voids in the morphology. Consequently, the effective current capacity is sharply reduced with the emergence of the porous structure. This is considered to be another barrier to obtaining high transport current in Mg^11^B_2_ wires. In addition, this kind of microstructure with abundant voids can be more brittle and thus be more prone to fracture and form big microcracks (see Fig. 4c) resulting from heat stress during the furnace-cooling process from high temperature to room temperature.

**Figure 5:**
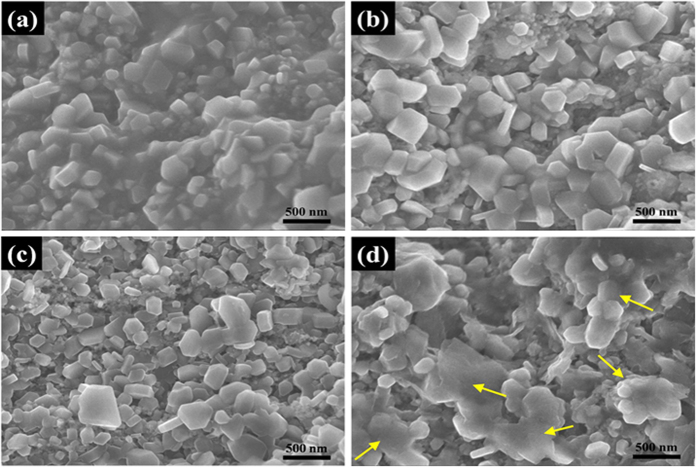
High-resolution SEM micrographs of longitudinal sections of Mg^11^B_2_ wires sintered at (**a**) 700 °C, (**b**) 750 °C, (**c**) 770 °C, and (**d**) 800 °C. The shapes of grains can be easily distinguished. The yellow arrows in (**d**) indicate clusters composed of multiple Mg^11^B_2_ grains.

High-resolution SEM was employed to investigate the details of the crystalline structure in the four Mg^11^B_2_, wires, and the results are presented in Fig. 5. In the sample with the lowest sintering temperature, the crystalline grains have a wide range of sizes, and all of them are dispersed in a melted matrix, as shown in Fig. 5(a). Referring to the XRD results above, the melted matrix is un-reacted Mg, which cannot be fully reacted with B at a relatively low temperature. This is strong evidence for the smaller mass fraction of Mg^11^B_2_ phase and lower transport performance in this sample. In the wires sintered at higher temperature, the amount of un-reacted Mg is greatly reduced, and the Mg^11^B_2_ crystalline grains keep growing and form typical hexagonal shapes, which can be observed in Fig. 5(b,c). Figure 5(d) shows the morphology of the wire sintered at 800 °C, in which some grains abnormally grow, and abundant big clusters are found. These clusters are formed by the localized aggregation of Mg^11^B_2_ grains at the relatively high heat-treatment temperature. This phenomenon further increases the porosity on the macroscale and significantly reduces the effective superconducting fraction for transporting current. As a result, the inter-grain connectivity is badly degraded. Combining these results with the low-magnification SEM images, it is thus concluded that the vanishing of transport current in the Mg^11^B_2_ sintered at high temperature should be attributed to the depression of inter-grain connectivity in the wire that is caused by the big microcracks and high porosity.

## Conclusions

The effects of sintering temperature on the superconducting performance and morphology of Mg^11^B_2_ monofilament wires made from isotopically pure boron powder were investigated in this work. It was found that increasing the sintering temperature led to the evolution of microstructure and characteristic changes in the transport current capacity. Un-reacted Mg and B-rich phase existed in the wire sintered at low temperature. The Mg^11^B_2_ fraction, as well as the transport performance, was reduced because of the un-reacted Mg and B-rich phase impurities. With increasing sintering temperature, better phase composition and crystallinity were obtained. The best transport *J*_*c*_ = 2 × 10^4^ A/cm^2^ was reached at 4.2 K and 5 T in the Mg^11^B_2_ wire sintered at 750 °C. It should be noted that although high magnetic *J*_*c*_ was detected in the wire sintered at 800 °C, the transport current was totally absent. The evolution of the morphology could be clearly seen in the wires corresponding to different sintering temperatures. Due to the abnormal growth and high mobility of Mg^11^B_2_ grains at relatively high ambient temperature, numerous big microcracks, voids, and Mg^11^B_2_ clusters formed in the wire sintered at 800 °C. As a result, the inter-grain connectivity was significantly suppressed, resulting in the inferior transport performance. The results obtained in our work can be a constructive guide for fabricating Mg^11^B_2_ wires to be used as magnet coils in fusion reactor systems such as ITER-type tokamak magnets.

## Additional Information

**How to cite this article**: Qiu, W. *et al*. Improvement in the transport critical current density and microstructure of isotopic Mg^11^B_2_ monofilament wires by optimizing the sintering temperature. *Sci. Rep.* **6**, 36660; doi: 10.1038/srep36660 (2016).
